# Osteostatin Inhibits M-CSF+RANKL-Induced Human Osteoclast Differentiation by Modulating NFATc1

**DOI:** 10.3390/ijms23158551

**Published:** 2022-08-01

**Authors:** Lidia Ibáñez, Josep Nácher-Juan, María Carmen Terencio, María Luisa Ferrándiz, María José Alcaraz

**Affiliations:** 1 Department of Pharmacy, Cardenal Herrera-CEU Universities, 46115 Valencia, Spain; lidia.ibanez@uchceu.es; 2 Interuniversity Research Institute for Molecular Recognition and Technological Development (IDM), Polytechnic University of Valencia, University of Valencia, 46100 Valencia, Spain; jojuana3@uv.es (J.N.-J.); carmen.terencio@uv.es (M.C.T.)

**Keywords:** PTHrP C-terminal peptides, osteostatin, bone, osteoclast, NFATc1

## Abstract

Parathyroid hormone-related protein (PTHrP) C-terminal peptides regulate the metabolism of bone cells. PHTrP [107–111] (osteostatin) promotes bone repair in animal models of bone defects and prevents bone erosion in inflammatory arthritis. In addition to its positive effects on osteoblasts, osteostatin may inhibit bone resorption. The aim of this study was to determine the effects of osteostatin on human osteoclast differentiation and function. We used macrophage colony-stimulating factor (M-CSF) and receptor activator of nuclear factor κB ligand (RANKL) to induce the osteoclast differentiation of adherent human peripheral blood mononuclear cells. Tartrate-resistant acid phosphatase (TRAP) staining was performed for the detection of the osteoclasts. The function of mature osteoclasts was assessed with a pit resorption assay. Gene expression was evaluated with qRT-PCR, and nuclear factor of activated T cells, cytoplasmic 1 (NFATc1) nuclear translocation was studied by immunofluorescence. We observed that osteostatin (100, 250 and 500 nM) decreased the differentiation of osteoclasts in a concentration-dependent manner, but it did not modify the resorptive ability of mature osteoclasts. In addition, osteostatin decreased the mRNA levels of cathepsin K, osteoclast associated Ig-like receptor (OSCAR) and NFATc1. The nuclear translocation of the master transcription factor in osteoclast differentiation NFATc1 was reduced by osteostatin. Our results suggest that the anti-resorptive effects of osteostatin may be dependent on the inhibition of osteoclastogenesis. This study has shown that osteostatin controls human osteoclast differentiation in vitro through the downregulation of NFATc1.

## 1. Introduction

Metabolic bone diseases are caused by an imbalance between bone formation by osteoblasts and bone resorption mediated by osteoclasts. The excess formation or activity of osteoclasts causes pathological bone resorption in conditions such as postmenopausal osteoporosis, rheumatoid arthritis, and periodontal disease. Furthermore, enhanced bone resorption and loss of calcium are skeletal complications associated with many cancers and bone metastases [1].

Parathyroid hormone-related protein (PTHrP) is a critical regulator of bone formation and repair. The N-terminal fragment PTHrP [1–36], which shows structural analogy with PTH [1–34], is an anabolic skeletal agent [2]. Both the N-terminal and the PTH-unrelated C-terminal PTHrP fragments may help to restore bone metabolism altered by glucocorticoids [3] and exert similar osteogenic effects in the appendicular skeleton of ovariectomized mice [4]. Several investigations have demonstrated that PTHrP C-terminal peptides stimulate osteoblast growth [5] and inhibit bone resorption [6,7]. Thus, the reported effects of PTHrP [107–139] may be dependent on its anabolic and anti-resorptive properties [4]. In particular, the activity of this peptide in bone cells may reside in the 107–111 sequence [5,8]. This pentapeptide PHTrP [107–111] (osteostatin) which includes the -Thr-Arg-Ser-Ala-Trp- sequence of the PTHrP C-terminal fragment is able to promote the osteogenic differentiation of mesenchymal stem cells [9], inhibit senescence and inflammation in osteoarthritic osteoblasts [10], reverse the skeletal alterations associated with insulin-like growth factor I deficiency [11] and improve bone regeneration in animal models of bone defects [12,13,14]. We have reported that osteostatin downregulates the immune response to collagen II and decreases the severity of arthritis in mice [15]. In addition to the immunomodulatory and anti-inflammatory actions, we observed that osteostatin prevented bone loss in this model of rheumatoid arthritis and reduced the area of tartrate-resistant acid phosphatase (TRAP) staining in mice ankle sections, suggesting a reduction in the number of osteoclasts in inflamed joints. 

Osteoclasts are multinucleated cells formed by the fusion of precursors in the myeloid/monocyte lineage. They are the principal resorptive cells of bone, and they play a central role in the regulation of bone mass [16]. The differentiation of osteoclasts mainly depends on receptor activator of nuclear factor κB (NF-κB)(RANK) ligand (RANKL) and macrophage colony-stimulating factor (M-CSF) produced by osteoblasts [17]. M-CSF binds to its receptor M-CSFR on early osteoclast precursors, providing signals for their survival and proliferation [16]. RANKL binds its receptor RANK on precursor cells and osteoclasts to exert key effects in differentiation, function and survival [18]. The activation of different signaling pathways by RANKL and M-CSF leads to the upregulation of the transcription factor nuclear factor of activated T cells cytoplasmic 1 (NFATc1), promoting the expression of osteoclast-specific genes and the differentiation of osteoclast precursor cells into mature osteoclasts [19]. Although a number of studies have suggested that osteostatin anti-resorptive activity is mediated by direct actions on osteoclasts [8,20], species differences and lack of activity on these cells have also been reported [21]. The aim of this study was to determine whether osteostatin can regulate the differentiation and function of human osteoclasts and the mechanism involved.

## 2. Results

### 2.1. Effect of Osteostatin on Osteoclast Differentiation

**Figure 1:**
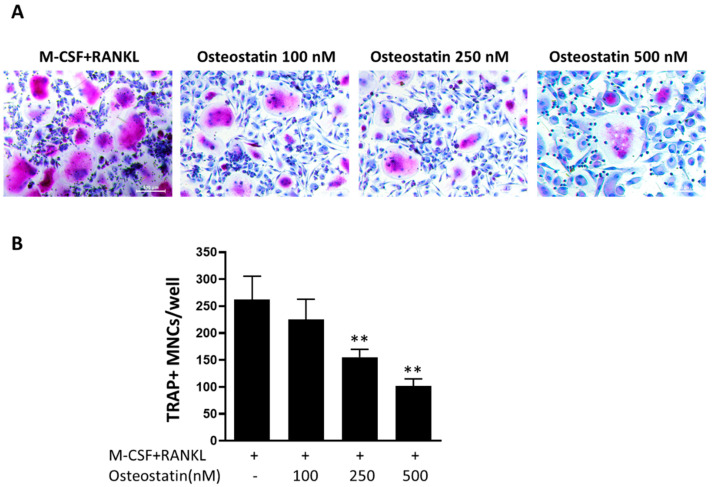
The effects of osteostatin on osteoclast differentiation. Osteoclast precursors were stimulated with M-CSF and RANKL for osteoclast differentiation in the presence or absence of osteostatin (final concentrations: 100, 250 and 500 nM) for 7–9 days. Cells were TRAP and hematoxylin stained, and TRAP^+^ positive cells with 3 or more nuclei were counted under a light microscope. (**A**) Representative images; Bar = 100 μm. (**B**) TRAP^+^ multinucleated cells (MNCs) per well are expressed as the mean ± S.D. of three independent experiments; ** *p* < 0.01 vs. M-CSF+RANKL.

We first examined the activity of osteostatin on osteoclast differentiation. For this purpose, adherent peripheral blood mononuclear cells (PBMCs) were cultured with M-CSF and RANKL to induce their differentiation into osteoclasts in the presence or absence of osteostatin, and then multinucleated TRAP^+^ cells were counted. Osteoclast precursors were exposed to osteostatin at the concentrations of 100, 250 and 500 nM. These concentrations did not exert any cytotoxic effect on these cells in previous experiments assessed using the MTT assay (data not shown). Figure 1 shows that osteostatin decreased the differentiation of osteoclasts in a concentration-dependent manner with statistically significant reductions in the number of TRAP^+^ multinucleated cells at 250 and 500 nM.

### 2.2. Osteoclast Functional Activity 

Mature osteoclasts are characterized by their ability to resorb mineralized matrix and form resorption pits. As osteostatin decreases osteoclast differentiation, to assess whether this peptide could affect osteoclast function, we performed the resorption assay using mature osteoclasts. That is, we first differentiated osteoclasts from human PBMCs and then seeded mature osteoclasts onto the Osteo Assay Surface 96-well plate.

**Figure 2:**
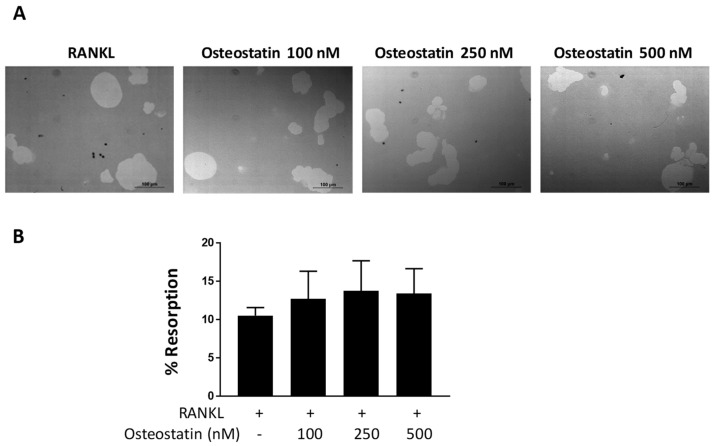
The effects of osteostatin on resorption. Differentiated osteoclasts were seeded on a 96-well osteoassay plate and incubated with RANKL in the presence or absence of osteostatin (final concentrations: 100, 250 and 500 nM) for 2 days. (**A**) Representative images of resorption pits; Bar = 100 μm. (**B**) Percentage of resorption area. Values are the mean ± S.D. of four independent experiments.

Cells were incubated with osteostatin at concentrations of 100, 250 and 500 nM, and resorption was analyzed. Image quantification of the resorption area indicated that this peptide did not significantly modify the resorption process induced by mature human osteoclasts in vitro (Figure 2).

### 2.3. Effect on Gene Expression

**Figure 3:**
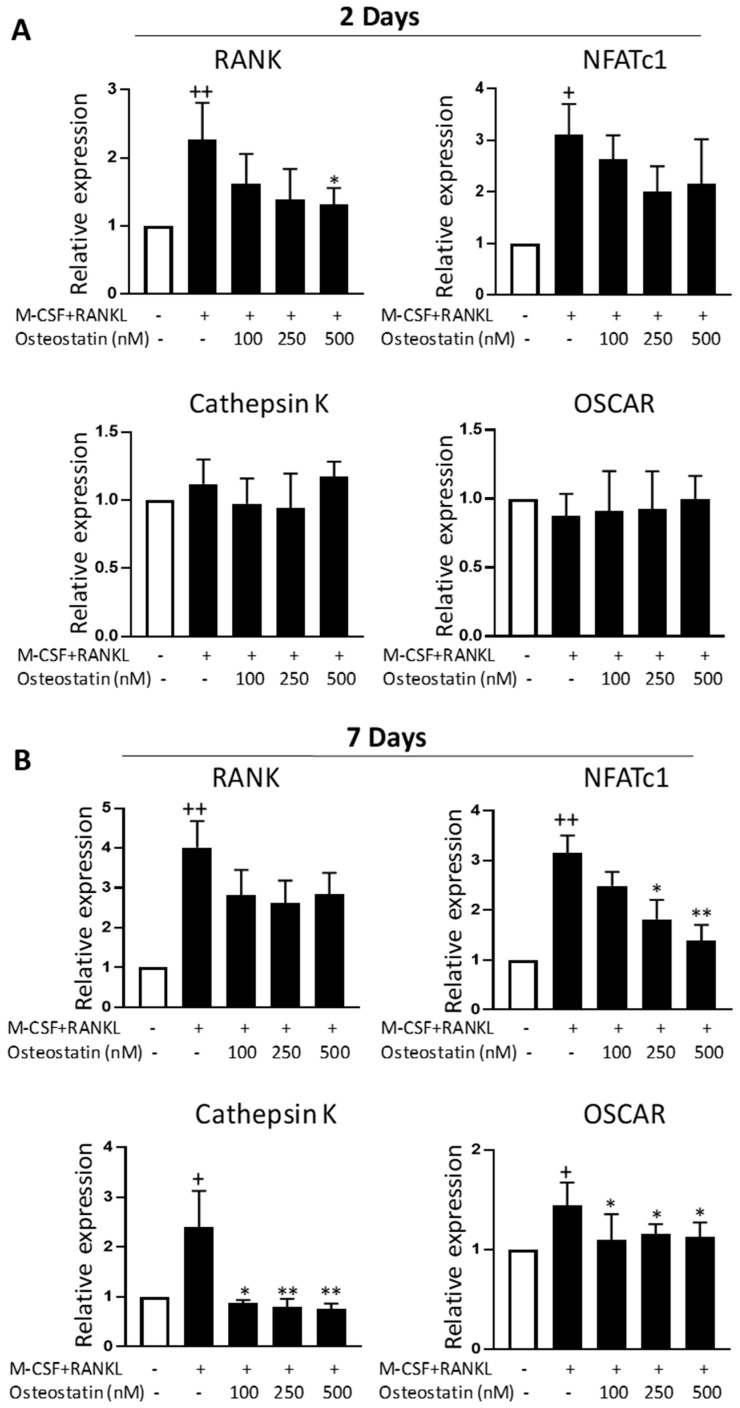
The effects of osteostatin on the mRNA expression of osteoclast markers. (**A**) Expression levels at 2 days of differentiation. (**B**) Expression levels at 7 days of differentiation. Osteoclast precursors were incubated with M-CSF and RANKL in the presence or absence of osteostatin (final concentrations: 100, 250 and 500 nM). The levels of mRNA expression were determined with qRT-PCR and normalized to glyceraldehyde-3-phosphate dehydrogenase (GAPDH). Values are the mean ± S.D. of four independent experiments; + *p* < 0.05, ++ *p* < 0.01 vs. control; * *p* < 0.05, ** *p* < 0.01 vs. M-CSF+RANKL.

The effects of osteostatin on gene expression were examined in osteoclast precursors stimulated by RANKL and M-CSF. We determined using qRT-PCR how osteostatin affects a set of osteoclast-associated genes during early (day 2) and late (day 7) osteoclast differentiation stages. As shown in Figure 3, M-CSF+RANKL induced the mRNA expression of RANK and NFATc1 at both time points, while the mRNA levels of cathepsin K and osteoclast associated Ig-like receptor (OSCAR) were enhanced by these cytokines at day 7. 

The treatment of differentiating osteoclasts with osteostatin slightly reduced the mRNA expression of the receptor RANK, which was statistically significant only at the highest concentration after 2 days of incubation (Figure 3A). The transcription factor NFATc1 is a master regulator of osteoclast differentiation [22]. Figure 3B shows that osteostatin dose-dependently decreased the expression level of NFATc1 mRNA at day 7 (late osteoclast differentiation stage). At this time point, osteostatin treatment also reduced the gene expression of osteoclast markers cathepsin K and OSCAR at the three concentrations tested.

### 2.4. NFATc1 Nuclear Translocation

**Figure 4:**
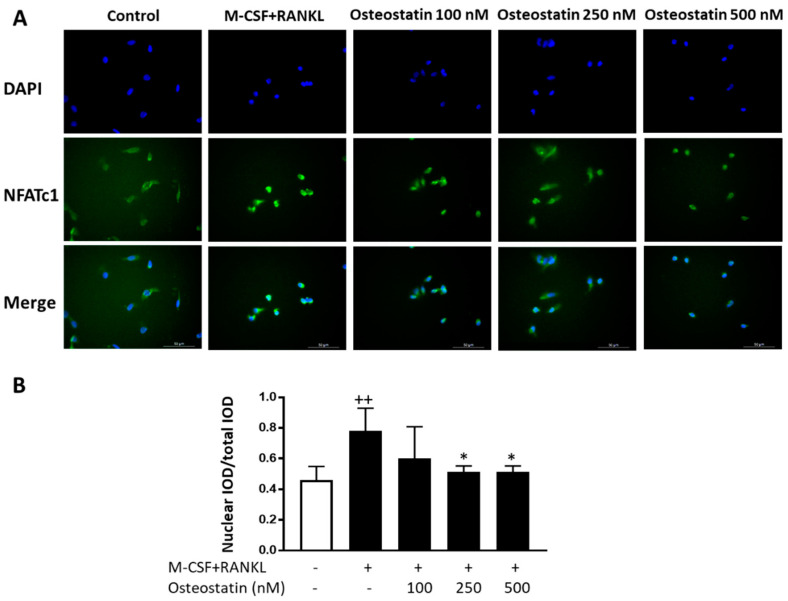
The effects of osteostatin on NFATc1 nuclear translocation. Osteoclast precursors were stimulated with M-CSF and RANKL in the presence or absence of osteostatin (final concentrations: 100, 250 and 500 nM) for 2 days, and then NFATc1 nuclear translocation was examined by immunofluorescence. (**A**) Representative images; Bar = 50 μm. Nuclei were stained by DAPI. (**B**) The nuclear/total integrated optical density (IOD) ratio was obtained to compare NFATc1 nuclear translocation. Results are expressed as the mean ± S.D. of three independent experiments; ++ *p* < 0.01 vs. control; * *p* < 0.05 vs. M-CSF+RANKL.

RANKL stimulation enhances NFATc1 expression and activation to induce genes involved in osteoclast differentiation. To gain insight into the mechanism of action of osteostatin, we studied the effects of this peptide on NFATc1 activation as this transcription factor is crucial for osteoclast differentiation. The expression of NFATc1 protein and its cellular localization were analyzed by immunofluorescence. Figure 4 shows that osteoclastogenic medium induced the translocation of NFATc1 from the cytoplasm to the nucleus during the early phase of differentiation into osteoclasts. Osteostatin treatment resulted in a concentration-dependent reduction in NFATc1 nuclear translocation, which was statistically significant at the concentrations of 250 and 500 nM.

## 3. Discussion

PTHrP C-terminal peptides including osteostatin have been suggested as novel agents with the ability to regulate bone metabolism and avoid bone loss. These molecules would have dual actions, promoting formation and inhibiting resorption to control bone homeostasis. A wide range of evidence supports their anabolic effects on bone [12,13,14], although some discrepancies have been reported concerning their anti-resorptive ability and their possible effects on osteoclasts. The C-terminal peptides chicken and human PTHrP [107–139] and osteostatin inhibited basal bone resorption by osteoclasts isolated from 15 day embryonic chickens [20], and osteostatin also reduced TRAP levels in cultured rat osteoclasts [23]. In addition, human PTHrP [107–139] inhibited basal and stimulated osteoclastic bone resorption, decreased the number of mononucleated osteoclast-like cells [7] and exerted anti-resorptive effects in vivo [24]. Nevertheless, another study failed to identify any significant effect of osteostatin or other PTHrP peptides on the resorptive activity of isolated rat or chick osteoclasts [21]. Additionally, human PTHrP [107–139] and osteostatin did not modify resorption in neonatal mouse calvaria in vitro [25]. Therefore, the actions of osteostatin and other related C-terminal PTHrP peptides on osteoclast activity and bone resorption are not fully understood. 

In vivo, some investigations support an anti-resorptive effect of osteostatin. Subcutaneous injection of 0.5 μg/kg of osteostatin at the same time of administration of PTHrP [1–34] (0.2 μg/kg) daily for 6 or 12 days prevented the resorptive effect of this peptide in neonatal mice [26], and we have reported that administration of 80 or 120 μg/kg/day of osteostatin inhibited bone erosion and reduced the osteoclast area in sections of ankles from collagen-induced arthritis mice [15], indicating the activity of this peptide in inhibiting inflammatory bone loss.

In the current study, we have used M-CSF and RANKL, which are cytokines critical for osteoclastogenesis [27], to induce the differentiation of human osteoclasts in vitro. We have also assessed the resorptive function of mature osteoclasts using a resorption pit assay. Our results show that osteostatin reduces the differentiation of human osteoclasts in vitro but it does not inhibit the resorptive function in differentiated osteoclasts, thus suggesting that the anti-resorptive effects of this peptide may be dependent on the inhibition of osteoclastogenesis.

M-CSF and RANKL drive the expression of a number of osteoclast-specific genes such as those encoding TRAP, cathepsin K, OSCAR and calcitonin receptor, leading to the development of mature osteoclasts [28,29]. Our data indicate that osteostatin decreases the mRNA expression of osteoclast markers, confirming the inhibitory effects of this peptide on osteoclast differentiation. 

As NFATc1 induction and activation by RANKL play an essential role in osteoclastogenesis [22], we have focused on osteostatin’s effects on this transcription factor. Activation of RANK by its ligand on osteoclast precursor cells induces the recruitment and activation of cytosolic tumor necrosis factor receptor-associated factors as adaptor molecules to activate signaling pathways such as NF-κB, activator protein-1 and mitogen-activated protein kinases, which are essential downstream targets of early signaling by RANK-RANKL. This leads to the activation of the NFATc1 promoter during the early phase of osteoclast differentiation. After this initial induction, RANK signaling cooperates with other systems to achieve a robust amplification of NFATc1 based on an autoamplification mechanism and the translocation of this transcription factor to the nucleus to upregulate the transcription of target genes necessary for osteoclast differentiation and function [22,30]. 

PTHrP is a ligand of the PTH type 1 receptor (PTH1R) in osteoblastic cells. This receptor is a member of the class B G protein-coupled receptors signaling through pathways such as classical cAMP/protein kinase A and phospholipase C β/intracellular calcium/protein kinase C (PKC) activation cascades [31]. Structural studies have mainly focused on the interaction of N-terminal fragments with PTH1R signaling, and little is known of the mechanisms of action of C-terminal peptides such as osteostatin. It is possible that C-terminal domains of PTHrP can function independently of PTHR1. Thus, the direct interaction of PTHrP [107–139] with PTH1R-unrelated receptors associated with an induced Ca^2+^ influx has been suggested in osteoblastic cells [32]. In osteoclasts, it has been reported that PTHrP [107–139] does not act via a cAMP signal transduction mechanism, but its effects may be mediated by PKC [6]. In addition, osteostatin may exert indirect effects on osteoclastogenesis in vivo through the inhibition of cytokine production by osteoblasts [10]. 

In this study, we found that osteostatin inhibited NFATc1 translocation to the nucleus during the early phase of osteoclast differentiation, which indicates that this peptide may inhibit the activation of this transcription factor. The consequence would be the downregulation of osteoclast-specific genes such as cathepsin K and OSCAR and of NFATc1 itself, as shown in the mRNA analysis, leading to the inhibition of osteoclast differentiation. Thus, our results indicate that osteostatin inhibits human osteoclastogenesis in vitro by suppressing NFATc1 activity.

In recent years, a better understanding of bone cell function and of the pathophysiology of bone conditions has led to the incorporation of new therapeutic agents. Nevertheless, as they can have relevant side effects and exert a partial protection, there are additional opportunities for developing new inhibitors of bone resorption and/or stimulators of bone formation [1]. In fact, it is possible that combination therapy with anti-resorptive and anabolic drugs would provide even greater benefits than either drug alone [33]. In this respect, osteostatin would combine both properties in the same molecule and may be of interest to investigate potential new treatments in bone diseases.

## 4. Materials and Methods

### 4.1. Osteoclast Differentiation from Human PBMCs and TRAP Staining

Fresh buffy coats of anonymous healthy donors were provided by the Blood Transfusion Center, Generalitat Valenciana (Valencia, Spain). The procedure was approved by the Committee of Ethics and Human Research, University of Valencia (No. H1512058528966). PBMCs were isolated by centrifugation over Ficoll-Paque™PLUS (Cytiva, Thermo Fisher Scientific, Madrid, Spain) and plated in cell-culture dishes in Roswell Park Memorial Institute (RPMI) medium (Gibco, Thermo Fisher Scientific) with 10% fetal bovine serum (FBS) (Hyclone, Thermo Fisher Scientific) and 1% penicillin–streptomycin (Gibco, Thermo Fisher Scientific). After 2 h of incubation (37 °C, 5% CO_2_), nonadherent cells were removed, and the adherent cells were collected and cultured in 96-well plates at 1 × 10^5^ cells/well in MEM-alpha (Gibco, Thermo Fisher Scientific) osteoclastogenic medium containing 10% FBS (Hyclone, Thermo Fisher Scientific), 1% penicillin-streptomycin (Gibco, Thermo Fisher Scientific), 25 ng/mL M-CSF (PeproTech, London, UK) and 50 ng/mL RANKL (PeproTech) either with or without osteostatin (Bachem AG, Bubendorf, Switzerland) at a final concentration of 100, 250 or 500 nM. Medium was changed every 3–4 days until fully differentiated osteoclasts were detected in the control. Then, cells were fixed with 4% paraformaldehyde (PFA) in phosphate-buffered saline (PBS) for 5 min, washed and incubated in 0.2 M acetate buffer (0.2 M sodium acetate, 0.05 M tartaric acid and water; pH 5.0) for 20 min at 37 °C. Cells were then washed and incubated in a solution containing distilled water, 0.5 mg/mL naphthol AS-MX phosphate (Sigma-Aldrich, Madrid, Spain), and 1.1 mg/mL fast red violet LB salt (Sigma-Aldrich) for 20 min at 37 °C. Finally, cells were washed and counterstained with hematoxylin. TRAP^+^ cells with ≥3 nuclei were counted using a DM IL LED microscope (Leica, Wetzlar, Germany) and a Leica DFC 450 C camera.

### 4.2. MTT Assay

The mitochondria-dependent reduction of 3-(4,5-dimethylthiazol-2-yl)-2,5 diphenyltetrazolium bromide (MTT) to formazan was assayed in adherent PBMCs cultivated as indicated above. Cells were then incubated with MTT (200 μg/mL, Sigma-Aldrich) for 2 h. Medium was removed, and cells were solubilized in dimethylsulfoxide (100 μL) to quantitate formazan at 550 nm using a Victor3 microplate reader (PerkinElmer, Madrid, Spain).

### 4.3. Resorption Assay 

Osteoclasts were differentiated as described above. When fully differentiated osteoclasts were detected, cells were collected using Accutase (Sigma-Aldrich), and a total of 10^4^ cells were seeded/well on an Osteo Assay Surface 96-well plate (Corning, Sigma-Aldrich) in MEM-alpha containing 10% FBS (Hyclone, Thermo Fisher Scientific) and 1% penicillin–streptomycin (Gibco, Thermo Fisher Scientific), with the medium supplemented with RANKL (30 ng/mL) (PeproTech) either with or without osteostatin at a final concentration of 100, 250 or 500 nM. After 48 h, cells were removed using distilled water. Imaging was performed using a DM IL LED microscope (Leica), and images were taken using the LAS software (Leica). The total areas of the resorption pits were determined using Fiji/ImageJ software [34]. 

### 4.4. Quantitative Reverse-Transcription PCR (qRT-PCR)

Osteoclast progenitors were cultured in MEM-alpha osteoclastogenic medium for 2 or 7 days. Then, total RNA was extracted from differentiating osteoclasts using the RNeasy plus mini kit (Qiagen AG, Hombrechtikon, Switzerland) according to the manufacturer’s instructions. Reverse transcription was accomplished on 0.5 μg of total RNA using random primers and a Transcriptor First Strand cDNA Synthesis Kit (Roche LifeScience. Basel, Switzerland). We amplified the sequences of interest using specific primers from Bio-Rad Laboratories (Hercules, CA, USA): PrimePCR™ SYBR Green^®^ Assays: qHsaCID0016934 (cathepsin K), qHsaCED0038674 (GAPDH), qHsaCID0023299 (NFATC1), qHsaCED0046751 (OSCAR) and qHsaCID0006213 (RANK) using SYBR Green PCR Master Mix (Bio-Rad) and an iCycler iQ™ Real-Time PCR Detection System (Bio-Rad). Differences in threshold cycle (ΔCt) values were calculated by correcting the Ct of the gene of interest to the Ct of the reference gene (GAPDH). Relative gene expression was expressed as 2^−ΔΔCt^ with respect to nonstimulated cells (control).

### 4.5. Immunofluorescence Analysis

Adherent PBMCs were collected, and a total of 10^5^ cells/well were seeded on a chamber slide (Nunc Lab Tek, Thermo Fisher Scientific) in MEM-alpha osteoclastogenic medium either with or without osteostatin at a final concentration of 100, 250 or 500 nM. After 48 h, cells were fixed with 4% PFA (10 min, RT), rinsed with PBS, treated with 5% Triton X-100 (10 min, RT) and thereafter incubated for 1 h with 1% BSA/PBS at RT. After rinsing, cells were incubated with the mouse anti-human antibody against NFATc1 (clone 7A6; Invitrogen, Thermo Fisher Scientific) (O/N, 4 °C). Goat anti-mouse IgGDyLight 488 (Invitrogen, Thermo Fisher Scientific) was used as a secondary antibody (1 h, RT). Slides were mounted in ProLong™ Gold Antifade reagent with 4′,6-diamidino-2-phenylindole (DAPI) (Molecular Probes, Thermo Fisher Scientific), and samples were analyzed under a fluorescence microscope (DM IL LED, Leica). Image acquisition was conducted with the LAS software (Leica), and cell fluorescence was measured using Fiji/ImageJ software [34]. NFATc1 nuclear translocation was quantified by measuring the area and fluorescent signal intensity of nuclear and total NFATc1. The DAPI staining mask was used to define the nuclear region of interest (ROI), and the total ROI is defined as the whole cell region (nuclear + cytoplasmatic areas). Nuclear and total integrated optical density (IOD) were calculated as area × intensity of fluorescence. Nuclear IOD/total IOD ratio was obtained to compare NFATc1 nuclear translocation. 

### 4.6. Statistical Analysis

All values are expressed as mean ± standard deviation (SD). Statistical significance was determined with one-way analysis of variance (ANOVA) with a post hoc Tukey’s test for multiple group comparisons. Data were analyzed using GraphPad Prism 7.0 (Graph Pad Software Inc., La Jolla, CA, USA). Results with *p* < 0.05 were considered statistically significant.

## Data Availability

All relevant data are presented in the manuscript. Raw data are available upon request from the corresponding author.
